# Comparison of Anticancer Activities and Biosafety Between *Salmonella enterica* Serovar Typhimurium ΔppGpp and VNP20009 in a Murine Cancer Model

**DOI:** 10.3389/fmicb.2022.914575

**Published:** 2022-06-29

**Authors:** Xiaoqing Liu, Yanxia Guo, Yujie Sun, Yu Chen, Wenzhi Tan, Jung-Joon Min, Jin Hai Zheng

**Affiliations:** ^1^School of Biomedical Sciences, Hunan University, Changsha, China; ^2^Department of Nuclear Medicine, Institute for Molecular Imaging and Theranostics, Chonnam National University Medical School and Hwasun Hospital, Hwasun, South Korea

**Keywords:** *Salmonella Typhimurium*, ΔppGpp, VNP20009, bacterial cancer therapy, biosafety

## Abstract

*Salmonella Typhimurium* defective in guanosine 5′-diphosphate-3′-diphosphate (ppGpp) synthesis (ΔppGpp) is an attenuated strain with good biosafety and excellent anticancer efficacy. It has been widely applied in preclinical studies of anticancer therapy for various types of solid cancer. VNP20009 is another genetically modified auxotrophic strain with 108-kb deletion, *purI^−^*, *msbB^−^*, and many single nucleotide polymorphisms (SNPs); it has shown promising therapeutic efficacy in various preclinical tumor models and entered phase I clinical trials. Here, the invasion activities and virulence of ΔppGpp were obviously lower than those of the VNP20009 strain when tested with cancer cells *in vitro*. In addition, the MC38 tumor-bearing mice showed comparable cancer suppression when treated with ΔppGpp or VNP20009 intravenously. However, the ΔppGpp-treated mice showed 16.7% of complete cancer eradication and prolonged survival in mice, whereas VNP20009 showed higher toxicity to animals, even with equal tumor size individually. Moreover, we found decreased levels of inflammatory cytokines in circulation but strengthened immune boost in tumor microenvironments of ΔppGpp-treated mice. Therefore, the engineered ΔppGpp has high potential for cancer therapeutics, and it is a promising option for future clinical cancer therapy.

## Introduction

Cancer is one of the major causes of rising mortality rates globally, and thousands of people suffer from malignant tumors every year. Among the different types of cancer, patients with colorectal cancer, liver cancer, lung cancer, and breast cancer show high mortality rates (Siegel et al., 2022; Xia et al., 2022). Radiotherapy, chemotherapy, targeted therapy, immunotherapy, and surgery are commonly used for cancer treatment, but with remarkable limitations retained, such as lack of tumor selectivity, limited tissue penetration, induction of anticancer drug resistance, and formidable side effects. Additionally, patients who receive conventional therapies have a high incidence of tumor recurrence. Bacteria-mediated cancer therapy (BMCT) was recognized in the nineteenth century when Busch and William B. Coley found that patients infected with *Streptococcus pyogenes* (McCarthy, 2006; Forbes et al., 2018) exhibit obvious tumor regression. With the development of molecular biology and the deep understanding of the tumor immune microenvironments, BMCT has once again aroused the interest of researchers as a promising approach for cancer therapy.

Hypoxia, vascular abnormalities, low pH, and necrotic areas are common features of solid tumors (Guo et al., 2020; Chen et al., 2021b; Kang et al., 2022), and they are the main barriers for traditional therapies. However, they provide favorable conditions for the targeting, colonization, and replication of some obligate or facultative anaerobes. Previous studies have shown that Bacillus Calmette–Guerin (Piao et al., 2022), *Clostridium* (Agrawal et al., 2004), *Listeria* (Kim et al., 2009), *Salmonella Typhimurium* (Paterson et al., 2010; Felgner et al., 2016), *Bifidobacterium* (Zhu et al., 2011), *Escherichia coli* (Kocijancic et al., 2016), and other bacteria manifest excellent antitumor effects. Among them, *S. Typhimurium* has been widely used in cancer treatment due to its specific tumor targeting ability and colonization to overcome the shortcomings of traditional cancer treatments, such as insufficient tumor selectivity and poor tissue penetration. Engineered *S. Typhimurium* has good biosafety, tumor targeting, and capacity to carry or express anticancer payloads, thereby providing suitable conditions for the combination of BMCT with other therapeutics (Guo et al., 2020; Al-Saafeen et al., 2021).

VNP20009 is an attenuated engineered *S. Typhimurium* with *purI* and *msbB* gene deletion, 108-kb deletion, and many single nucleotide polymorphisms (SNPs) with increased tumor chemotaxis and lowered tumor necrosis factor α (TNF-α)-induced sepsis. It is sensitive to various antibiotics and does not cause severe systemic toxicity upon infection (Zhang et al., 2015). Thus, VNP20009 has been safely used in a phase I clinical trial in patients with metastatic melanoma and renal cell carcinoma (Toso et al., 2002; Chorobik et al., 2013). Bacterial colonization was observed in tumor tissues, but the therapeutic efficacy needs to be further improved. The insufficient anticancer activity in clinical studies could be due to limited bacterial colonization in tumors and obvious side effects with increasing dosages. Another attenuated strain of *S. Typhimurium* defective in 5′-diphosphate-3′-diphosphate (ppGpp) synthesis (ΔppGpp) with the gene deletion of *relA* and *spoT* significantly increases the half-lethal dose of mice by 100,000–1,000,000-fold, and the functional loss of *Salmonella* pathogenicity island 1 (SPI-1) and SPI-2; it can serve as an ideal vector for targeted delivery and expression of oncolytic payloads (Zheng et al., 2017; Tan et al., 2022). ΔppGpp has also been used in molecular imaging for visualized tumor therapy due to its excellent tumor-specific colonization and low adverse effects (Jiang et al., 2013; Xiong et al., 2020; Wu et al., 2021).

In this study, we systemically studied ΔppGpp for its anticancer activity and biosafety in MC38-bearing mice, and we used the clinically tested VNP20009 as a control. Further analysis in ΔppGpp- or VNP20009-colonized MC38 tumors was performed to study the immune responses elicited by each attenuated *S. Typhimurium*. Finally, we evaluated bacteria-induced systemic toxicity in MC38-bearing mice after intravenous injection of ΔppGpp or VNP20009.

## Materials and Methods

### Cell Lines and Bacterial Strains

Murine colorectal cancer cell line MC38 was kindly provided by Dr. Je-Jung Lee (Chonnam National University, South Korea). Mouse melanoma B16F10, human colorectal adenocarcinoma Caco-2, and murine fibroblast NIH3T3 cells were obtained from the American Type Culture Collection (ATCC, CRL-6475, HTB-37, and CRL-1658). All the cells were cultured in Dulbecco’s modified Eagle medium (DMEM) containing 10% fetal bovine serum (FBS), 100 IU/ml penicillin, and 100 mg/ml streptomycin and incubated in a 37°C incubator containing 5% CO_2_. All experiments were performed with mycoplasma-free cells. Attenuated *S. Typhimurium* VNP20009 and ΔppGpp were derived from *S. Typhimurium* 14028S. Both strains were cultured in LB medium. Single colonies were cultured overnight in a shaking incubator at a constant temperature of 37°C and activated with fresh medium for 4 h the next day (1:100). Bacteria were harvested by centrifugation at 6,000 rpm for 2 min, diluted in PBS (pH 7.4), and measured with a UV spectrophotometer at OD_600_ (1 OD = 8 × 10^8^ CFU/ml).

### Bacterial Invasion Assay

Caco-2, NIH3T3, and MC38 cells were seeded in 24-well plates with a density of approximately 1 × 10^5^ cells per well and incubated overnight. The culture media were removed, and the cells were washed twice with Dulbecco’s PBS (DPBS), and DMEM (basic medium without additional supplement, 500 μl per well) was added for further culture. After overnight culture, VNP20009 and ΔppGpp were activated in fresh LB medium for 4 h. The bacteria were collected and washed twice with DPBS, and the bacterial number was quantified by measuring OD_600_ with a UV spectrophotometer (1 OD = 8 × 10^8^ CFU/ml). VNP20009 and ΔppGpp were used for cell infection with multiplicity of infections (MOIs) at MOI 10 and MOI 100, respectively. After 1 h of bacterial infection, the medium was removed, and the cells were washed with DPBS twice. To quantify the intracellular bacteria, DMEM containing 150 μg/ml gentamycin was added for an additional 30 min of culture. The medium was removed, and the cells were washed three times with DPBS. Subsequently, cell lysates were prepared with 0.05% Triton-X100 in PBS, diluted, and spotted on LB plates (dilution factor: 10^0^–10^3^). The bacteria were cultured in a 37°C incubator overnight, counted, and calculated the next day based on the dilution factors.

### Crystalline Violet Staining

MC38 and B16F10 cells were seeded in six-well plates at a density of approximately 2 × 10^4^ cells per well in six-well plates and incubated overnight. The next day, the culture medium was removed, the cells were washed twice with DPBS, and added with DMEM containing 10% FBS. VNP20009 and ΔppGpp were used to infect the cells at MOI 100. After 6 h of co-culture, the supernatants were removed and washed twice with DPBS. Complete DMEM culture medium (10% FBS, 100 IU/ml penicillin, and 100 mg/ml streptomycin) containing 150 μg/ml gentamycin was added to continue the culture. After 48 h, the cells were washed twice with DPBS, and 4% PFA was added to fix the cells at room temperature for 10 min. After 4% PFA was removed and the cells were washed twice with DPBS, 1 ml of 0.1% crystal violet dye was added and incubated at room temperature for 30 min. The crystal violet was recovered and washed twice with DPBS, and photographs were obtained and analyzed.

### Lactate Dehydrogenase Assay

MC38 and B16F10 cells were seeded in 24-well plates at a density of approximately 1 × 10^4^ cells per well and incubated overnight. VNP20009 and ΔppGpp were infected with MOI 100 for 6 h, and 150 μg/ml gentamycin was added for another 12 h of culture. The cell culture supernatants were examined with LDH assay (CytoTox 96 Non-Radioactive Cytotoxicity Assay) in accordance with the manufacturer’s instructions. The absorbance of the sample at 490 nm was detected using a microplate reader (Molecular Devices, SpectraMax M2/M2e, United States), and the release of LDH was calculated.

### Mice

Six-week-old female C57BL/6 mice were purchased from Hunan SJA Animal Company (Changsha, China). All mice were raised in a specific pathogen-free environment with a standard 12-h light/dark diurnal restriction. Sterilized water and food were given *ad libitum*. All experiments and euthanasia procedures were performed in accordance with protocols approved by the Hunan University Animal Research Committee (Changsha, China).

### Tumor Models and Management

Mice were shaved and placed under anesthesia in an induction box containing 2.5% isoflurane. MC38 (1 × 10^6^) cells were subcutaneously implanted into the right flank of the mouse. When the tumor size grew to 100–120 mm^3^, the bacteria were administered *via* tail vein injection. From the day of injection (day 0), the tumor sizes were measured and recorded every 3 days until the tumor volumes were ≥1,500 mm^3^. The mice were euthanized thereafter. In another set of experiments, the mice were killed on days 1, 3, and 7; the serum, tumor, and normal organs were collected for further analysis.

### Bacterial Viable Counts

To quantify the number of bacteria within the tumor tissue, tumors were excised from mice and homogenized in PBS. Samples were serially diluted (tenfold) and plated on LB agar plates. After overnight incubation at 37°C, the bacterial titer (CFU/g tissue) was determined by counting colonies and calculated with dilution factors and tissue weight.

### ELISA and Clinical Parameter Analysis

Serum and tumor tissues were separated from mice. After homogenization, protein lysates were collected from the supernatant after multiple centrifugations. The expression levels of IL-1β, TNF-α, IFN-γ, TGF-β, and IL-18 in the serum and supernatants of the tumor tissue homogenates were detected by ELISA kits in accordance with the manufacturer’s protocol (eBioscience). The clinical chemistry parameters, such as aspartate aminotransferase (AST), alanine aminotransferase (ALT), blood urea nitrogen (BUN), creatinine (CREA), C-reactive protein (CRP), and procalcitonin (PCT), in mouse sera were analyzed with an automatic biochemical analysis machine (Mindray BS-860, China).

### Immunofluorescence Analysis

Tumors isolated from mice were immersed in 4% PFA at 4°C for 2 h, immersed into 30% sucrose solution at 4°C overnight, embedded in tissue fixation solution (OCT), and rapidly frozen at −80°C. The neutrophil (Neu, Santa Cruz Biotech., SC-71674), *S. Typhimurium* (SL, Bio-Rad, 8,209–4,006), monocyte/macrophage (MOMA-2, Santa Cruz Biotech., SC-59332), and CD206 (M2 macrophage marker, Santa Cruz Biotech., SC-34577) in the tumor tissue sections of mice were analyzed by a standard immunofluorescence staining protocol. Images were obtained under an LSM510 fluorescence microscope (ZEISS, Germany) and processed using laser scanning microscopy (LSM) image software.

### Hematoxylin–Eosin Staining

Organs (heart, liver, spleen, lung, and kidney) isolated from mice were immersed in 4% PFA at 4°C for 2 h. The tumor tissues were transferred to 30% sucrose solution at 4°C overnight and embedded in tissue fixation solution (OCT) for rapid freezing at −80°C. After slicing with a cryotome, the slides underwent H&E staining following the standard protocol.

### Statistics

Statistical analysis was performed using GraphPad Prism 5.0 software. Mann–Whitney *U* test was used to determine the statistical significance of differences in tumor growth, cytokine expression, and changes in clinical chemical parameters between the control and treatment groups. A value of *p* < 0.05 was considered statistically significant. Survival analysis was performed using the Kaplan–Meier method and log-rank test. All data were expressed as means ± SEM.

## Results

### ΔppGpp Exerted Less Cytotoxicity *in vitro* Than VNP20009

**Figure 1:**
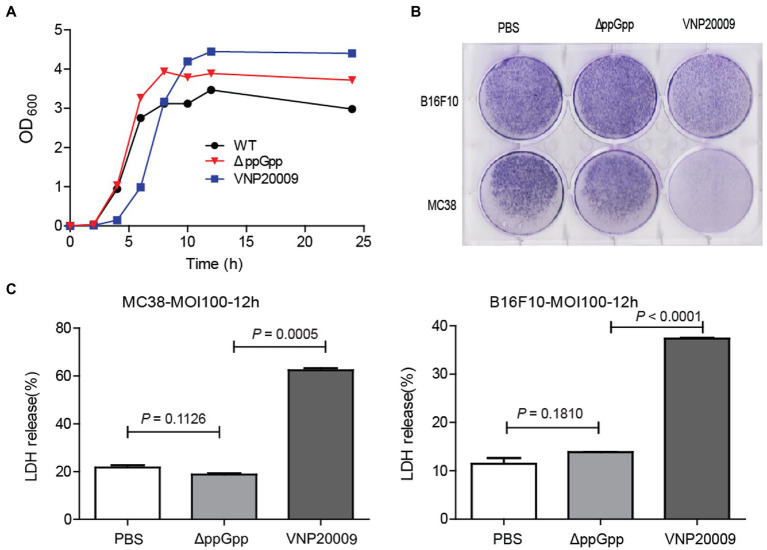
ΔppGpp showed weaker cytotoxicity than VNP20009 *in vitro*. **(A)** At 0, 2, 4, 6, 8, 10, 12, and 24 h, OD_600_ of ΔppGpp, VNP20009, and control group (WT) was measured with a UV spectrophotometer, and the growth curves of the three strains were drawn. **(B)** MC38 and B16F10 were co-cultured with ΔppGpp and VNP20009 for 6 h, respectively. After removing bacteria and changing basic DMEM with 150 μg/ml gentamycin for another 48 h of culture, crystal violet staining was performed to check the viable cells. **(C)** The LDH released from MC38 and B16F10 cells after co-culture with ΔppGpp and VNP20009 was determined with LDH assay, *p* = 0.0005 between ΔppGpp and VNP20009 in MC38 and *p* < 0.0001 between ΔppGpp and VNP20009 in B16F10.

The growth rate of *E. coli* is not affected by guanosine 5′-diphosphate-3′-diphosphate deficiency (Gaal and Gourse, 1990). Therefore, the growth of the mutants VNP20009 and ΔppGpp was measured, and growth curves were plotted (Figure 1A). The proliferation of these two mutants showed no significant difference compared with WT, indicating that the deficiency of certain genes did not affect bacterial growth, even with extended lag phase for VNP20009. The two strains were co-cultured with MC38 and B16F10 at MOI 100 in DMEM without supplement, respectively, to explore the cytotoxicity of mutant bacteria *in vitro*. At 6-h post-bacterial infection, the cells were continuously cultured in the medium containing 150 μg/ml gentamycin for another 48 h. The crystal violet staining results showed that the number of survived cells in the VNP20009-infected group was dramatically reduced, whereas no significant difference was found between the ΔppGpp-infected group and the PBS-treated control group (Figure 1B). LDH is an extremely stable cytoplasmic enzyme that exists in the cytoplasm of intact cells. It is released extracellularly once the cell membrane is damaged. The amount of LDH released after bacterial infection was measured to investigate the cell integrity affected by ΔppGpp and VNP20009 (Figure 1C). The results showed that the VNP20009-treated cells significantly increased LDH release in MC38 and B16F10 cells (*p* = 0.0005 in MC38 and *p* < 0.0001 in B16F10 versus no treatment or ΔppGpp-infected cells). No significant difference was found in the LDH levels between the ΔppGpp-infected group and the control group. These results indicated that ΔppGpp was much less toxic than the VNP20009 strain *in vitro*, and guanosine 5′-diphosphate 3′-diphosphate synthesis defect could further reduce the cytotoxicity of *S. Typhimurium*.

### ΔppGpp Showed Impaired Cell Invasion *in vitro*

**Figure 2:**
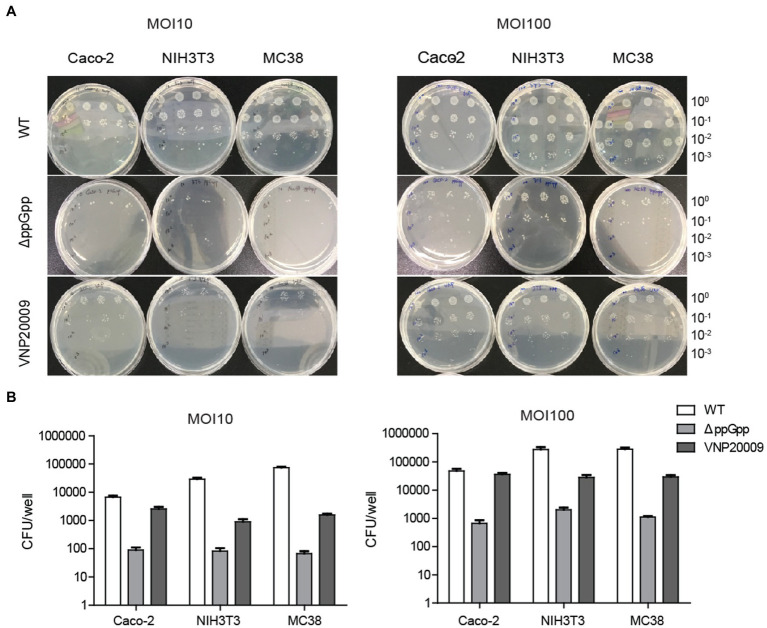
ΔppGpp showed weaker cell invasion than VNP20009 *in vitro*. **(A)** Caco-2, NIH3T3, and MC38 were co-cultured with ΔppGpp, VNP20009, and WT at different infection coefficients (MOI 10 and MOI 100) for 1 h, followed by 150 μg/ml gentamycin for an additional 30 min. The cells were then lysed to quantify the number of bacteria in the cells. **(B)** The numbers of bacteria invading cells under different infection conditions were recorded and analyzed.

Numerous Gram-negative bacteria are reported to infect host cells that are dependent on the type III secretion system (Ruano-Gallego et al., 2021; Sanchez-Garrido et al., 2021). This system is associated with bacterial invasion and intracellular growth, thereby affecting the efficiency of bacterial clearance and initial transient localization (Kang et al., 2022). Given that invasion ability is one of the important factors determining bacterial cytotoxicity, the invasion ability of VNP20009 and ΔppGpp to cells was compared *in vitro*. ΔppGpp and VNP20009 were co-cultured with Caco-2, NIH3T3, and MC38 cells at different infection coefficients (MOI 10 or MOI 100) for 1 h in basic DMEM and then cultured in medium containing 150 μg/ml gentamycin for another 30 min. The medium was removed, and the cells were washed three times with DPBS and lysed for bacterial quantification. As shown in Figures 2A,B, under the conditions of different infection coefficients, the intracellular bacteria of ΔppGpp in Caco-2, NIH3T3, and MC38 were significantly lower than that of the VNP20009 group and the WT control group. The results indicated that the cell invasion of ΔppGpp with the gene deletion of *relA* and *spoT* was significantly lower than that of VNP20009. Thus, the low bacterial burden should reduce the cytotoxicity of ΔppGpp to mammalian cells. This phenomenon could be a key factor to minimize damage to normal tissues and improve biosafety prior to bacterial colonization in tumors when administered *in vivo* for bacterial cancer therapy.

### Tumor Suppression and Bacterial Colonization in MC38 Xenograft Mice

**Figure 3:**
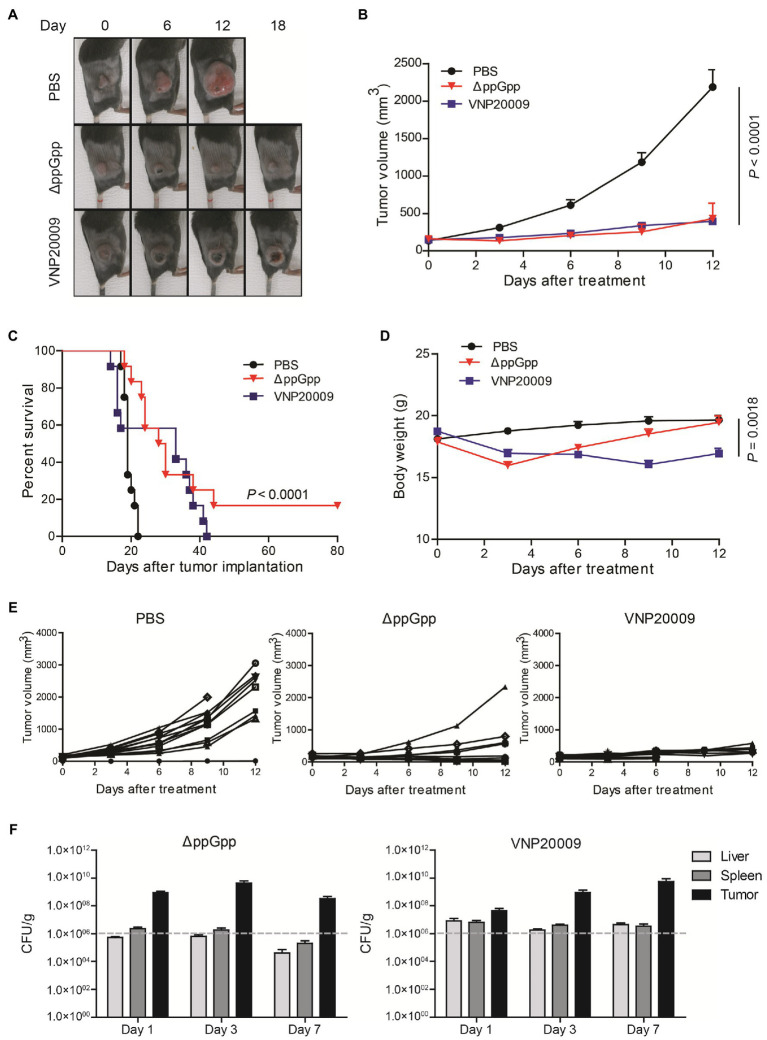
Comparison of VNP20009 and ΔppGpp in terms of animal survival and weight loss. **(A)** Photographs of subcutaneous tumors in representative mice. **(B)** Tumor growth curve in MC38-bearing mice, *n* = 12, *p* = 0.0001. **(C)** Animal survival curve of control, ΔppGpp-, and VNP20009-injected groups, *n* = 12, *p* < 0.0001 between PBS and ΔppGpp. **(D)** Change in body weight of mice after bacterial injection, *n* = 12, *p* = 0.0018 between PBS and VNP20009. **(E)** Tumor size change from individual mice. **(F)** Bacterial distribution in MC38-bearing mice at 1-, 3-, and 7-day post-bacterial injection, *n* = 8.

Previous studies have shown that engineered *S. Typhimurium* has robust anticancer activities due to its good tumor-specific targeting ability and proliferation (Liang et al., 2019). To investigate the anticancer activity of ΔppGpp *in vivo*, a MC38 murine colorectal cancer xenograft model was established. As shown in Figure 3A, tumor growth was obviously inhibited in the ΔppGpp- and VNP20009-injected groups compared with the control group (*p* < 0.0001). *Salmonella Typhimurium* treatments significantly delayed tumor growth, but no significant difference could be observed in the average tumor volumes between ΔppGpp and VNP20009 (Figure 3B). Even though overall tumor suppression was similar, the individual mouse between the two strain-treated groups was quite different. All the mice in the control and VNP20009-injected groups died or met the criteria for euthanasia (tumor volume ≥1,500 mm^3^) by approximately days 20 and 40, respectively, whereas 16.7% (2/12) of the ΔppGpp-injected mice exhibited eradicated tumor and survival after over 80 days of observation (Figure 3C; *p* < 0.0001). This finding indicated that ΔppGpp improved the survival of tumor-bearing mice. In addition, compared with the control group, the VNP20009-injected mice showed significant weight loss without recovery during the observation, whereas the ΔppGpp-injected mice rapidly regained their weight to normal (Figure 3D; *p* = 0.0018). Interestingly, VNP20009 showed an equal response in individual mice, whereas ΔppGpp showed a good response and variant anticancer activities among each mouse (Figure 3E). These results suggested that ΔppGpp has promising antitumor efficacy with excellent biosafety in the MC38 mouse model, which presented high animal survival and low body weight loss. However, further strategies should be applied to stabilize and enhance the anticancer activity of ΔppGpp. The bacterial distribution also revealed comparable bacterial colonization of both strains in tumor tissues, but enduring bacterial colonization in liver and spleen was observed in the VNP20009-treated group (Figure 3F).

### Cytokine Production Upon Bacterial Infection

**Figure 4:**
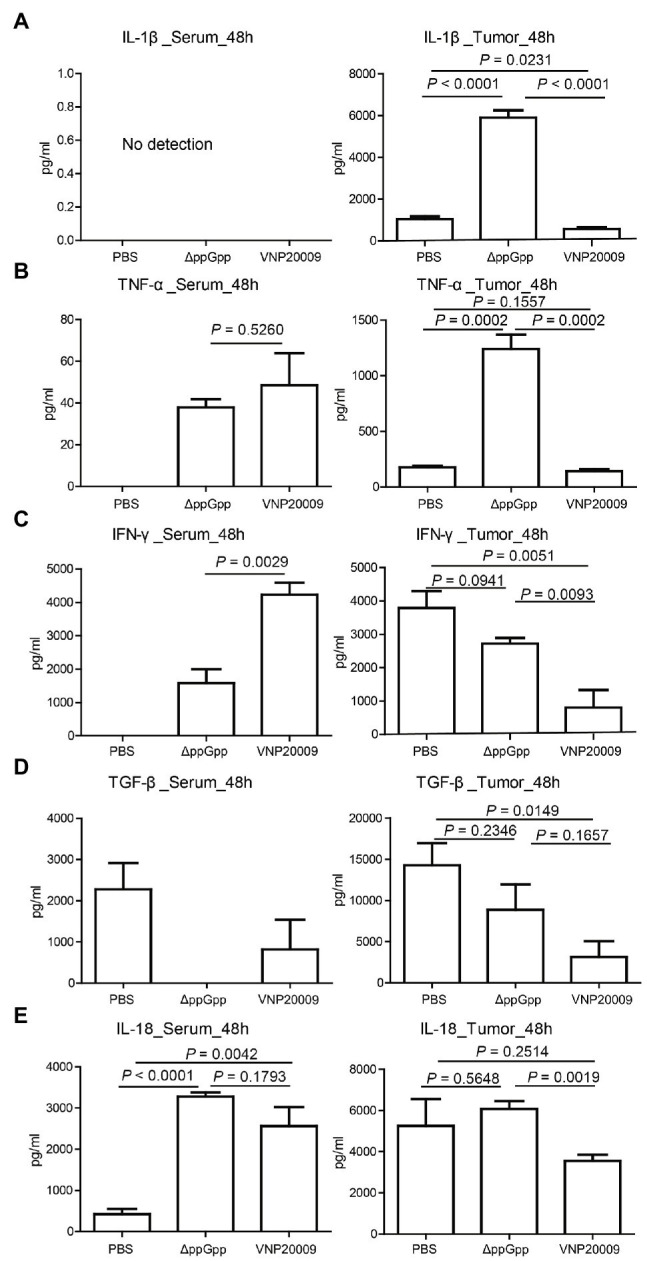
Cytokine production *in vivo* post-bacterial infection. **(A)** Determination of IL-1β expression in serum and tumor tissues of MC38-bearing mice at 48 h after bacterial injection (*n* = 4, *p* < 0.0001) between ΔppGpp and VNP20009 or PBS in tumor. **(B)** Expression of TNF-α in serum and tumor tissues of tumor-bearing mice after 48 h of bacterial injection (*n* = 4, *p* = 0.0002) between ΔppGpp and VNP20009 or PBS in tumor. **(C)** IFN-γ expression levels in serum and tumor tissues, *p* = 0.0029 between ΔppGpp and VNP20009 in serum, *p* = 0.0015 between PBS and VNP20009 in tumors, and *p* = 0.0093 between ΔppGpp and VNP20009 in tumors. **(D)** TGF-β in serum and tumor tissues at 48 h after bacterial injection, *n* = 4, *p* = 0.0149 between PBS and VNP20009. **(E)** IL-18 levels in serum and tumor tissues 48 h after bacterial injection, *n* = 4, *p* < 0.0001 between PBS and ΔppGpp, *p* = 0.0042 between PBS and VNP20009 in serum, and *p* = 0.0019 between ΔppGpp and VNP20009 in tumors.

Studies have shown that cytokine production plays an important role in maintaining immune balance and regulating pathological processes, such as immunological diseases and cancers (Bonati and Tang, 2021; Lim and Yoon, 2021). Bacterium-conserved components are strong stimulators for immune activation (Pangilinan and Lee, 2021). *Salmonella Typhimurium* infection can recruit abundant immune cells, such as neutrophils, macrophages, dendritic cells (DCs), and CD8^+^ T cells into the tumor microenvironment (TME); it can also induce the expression of pro-inflammatory cytokines to enhance the antitumor immune response (Cheng et al., 2014; Kim et al., 2015). In the present study, the cytokine levels in serum and tumor at 48-h post-bacterial injection were analyzed to determine the immune activation triggered by ΔppGpp and VNP20009. The expression of IL-1β in serum was not detected at 48 h after bacterial injection, but the level of IL-1β in tumor tissue was significantly higher in the ΔppGpp-injected group than in the VNP20009-injected and control groups (Figure 4A; *p* < 0.0001). In addition, the TNF-α levels in the tumor tissues of mice injected with ΔppGpp significantly increased, which may be one of the vital reasons for the antitumor activity of ΔppGpp (Figure 4B; *p* = 0.0002 versus the control and VNP20009-treated groups). The IFN-γ levels in the tumor tissues showed no significant difference between the ΔppGpp-injected and control groups, but both were significantly higher than that in the VNP20009-injected group (Figure 4C; *p* < 0.01). The TGF-β levels only significantly decreased in the VNP20009-injected group compared with those in the control group (Figure 4D; *p* = 0.0149). The IL-18 levels in tumor tissues from the ΔppGpp-injected group were also significantly higher than those in the VNP20009-injected group. No significant difference was found in the serum between the ΔppGpp- and VNP20009-treated groups, but they were both significantly higher than that in the control group (Figure 4E; *p* < 0.0001 versus ΔppGpp-treated group and *p* = 0.0042 versus VNP20009-treated group). These results showed that ΔppGpp and VNP20009 increased the inflammatory response in TMEs, thereby inducing a strong antitumor immune response. However, ΔppGpp showed more robust activation of immune response in MC38 tumor-bearing mice than VNP20009, with increased inflammatory cytokines in TMEs to relieve immunosuppression and reduce cytokine storm in circulating blood. Thus, ΔppGpp had better systemic biosafety than VNP20009.

### Immune Cell Infiltration in Tumor Tissues

**Figure 5:**
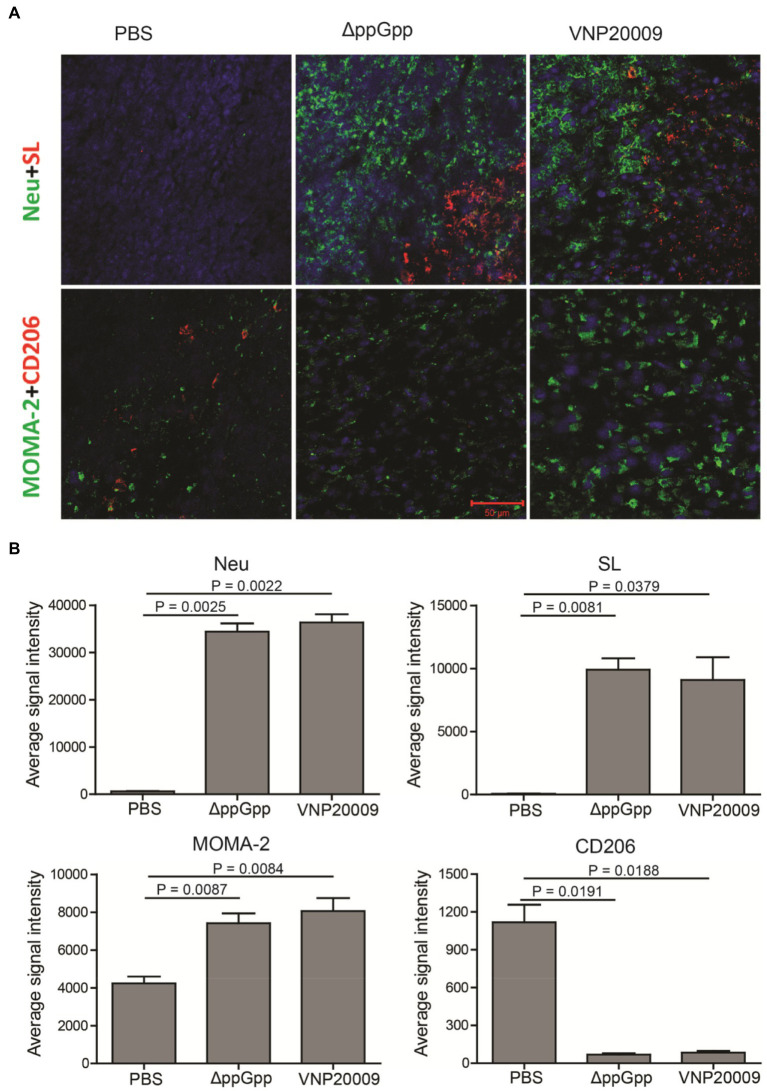
Bacterial colonization and immune cell analysis in TMEs. **(A)** Three days after bacterial injection, immunofluorescence staining was performed on the tumor tissues of neutrophil (Neu, green), *S. Typhimurium* (SL, red), monocyte/macrophage (MOMA-2, green), and M2 macrophage (CD206, red). Scale bar = 50 μm. **(B)** Quantification of the fluorescence intensity of neutrophils (Neu), *Salmonella* (SL), monocytes and macrophages (MOMA-2), M2-like macrophages (CD206), *n* = 4.

Previous studies have shown that *S. Typhimurium* can activate host anticancer immunity and recruit immune cells, including macrophages, natural killer (NK) cells, CD8^+^ T cells, and B cells (Lee et al., 2008; Kaimala et al., 2018). Mouse tumor tissues were analyzed in the present study by immunofluorescence staining. Extensive *S. Typhimurium* colonization was found in the tumor tissues in both ΔppGpp- and VNP20009-infected groups. This result was consistent with the bacterial quantification with Figure 3F. In addition, a huge amount of neutrophils and monocytes were recruited in the tumor tissues in the bacterium-injected group, accompanied with decreased M2-like macrophages (Figure 5A). Therefore, ΔppGpp and VNP20009 could effectively target and colonize tumor tissues and recruit immune cells to improve the tumor immune microenvironment. In addition to its intrinsic antitumor effects, attenuated *S. Typhimurium* could strongly promote tumor degeneration by activating the complex immune cells in TMEs (Pangilinan and Lee, 2021). Activation of immune cells in TMEs could relieve local immunosuppression and improve antitumor immunity. The quantification of the immunofluorescence staining signal also revealed significantly increased infiltration of neutrophils, monocytes, and macrophages, and significantly reduced M2-like macrophages post-bacterial injection. However, no significant difference was observed between ΔppGpp- and VNP20009-treated groups (Figure 5B). These results suggested that engineered bacteria have high potential of immune cell recruitment and further modification of TMEs to enhance the efficacy of immunotherapy.

### Biosafety Analysis of Mutant Strains

**Figure 6:**
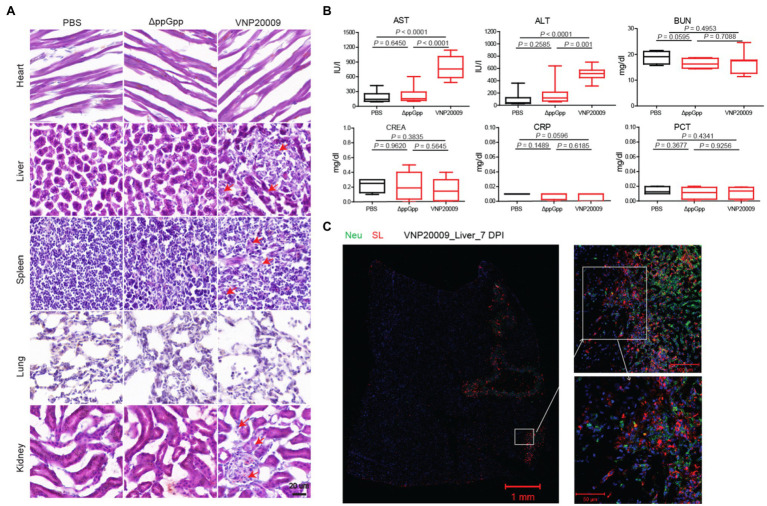
Biosafety studies after ΔppGpp and VNP20009 treatments. **(A)** After 7 days of bacterial injection, organs (heart, liver, spleen, lung, and kidney) from treated mice were analyzed by H&E staining. Scale bar = 20 μm. **(B)** Measurement of clinical chemical parameters, such as ALT (*p* < 0.0001), AST (*p* < 0.0001 between PBS and VNP20009 and *p* = 0.0001 between ΔppGpp and VNP20009), BUN, CREA, CRP, and PCT, in mouse serum 7 days after bacterial injection (*n* = 8). **(C)** Seven days after bacterial injection, the neutrophils (green) and *S. Typhimurium* (red) in the liver of VNP20009-injected mice were analyzed by immunofluorescence staining.

ΔppGpp and VNP20009 were injected into a MC38 xenotransplantation mouse model through tail vein injection. To further evaluate the tissue damage caused by the engineered strains, the organs were removed for frozen section at 7-day post-bacterial injection. H&E staining was carried out in accordance with the standard protocols. The results showed no significant change between the heart and lung in the VNP20009- and ΔppGpp-injected groups and the PBS control group. However, as shown in Figure 6A, the injury of liver and spleen in the VNP20009-injected group was obviously greater than that in the ΔppGpp-injected group and the control group, whereas ΔppGpp showed no significant difference from the control group. In addition, the renal pelvis of mice treated with VNP20009 was much smaller than that of the two other groups, suggesting the functional loss of the kidney. These results indicated that the ΔppGpp strain exerted less damage to the organs of tumor-bearing mice than VNP20009. Clinical chemical parameters in serum were checked to further study the biosafety, including ALT (17–77 IU/L), AST (54–298 IU/L), BUN (8–33 mg/dl), CREA (0.2–0.9 mg/dl), CRP (<0.5 mg/dl), and PCT (<0.5 ng/ml). The results showed no significant change in BUN, CREA, CRP, and PCT among the three groups. However, ALT and AST in the serum of the VNP20009-injected group were obviously higher than those of the ΔppGpp-injected and control groups, whereas the clinical chemical parameters in the serum of the ΔppGpp-injected group were not significantly different from those of the control (Figure 6B). At 7 days after VNP20009 injection, immunofluorescence staining was performed in the liver of VNP20009-treated mice because obvious damage was observed. The results showed extensive bacterial colonization and neutrophil infiltration (Figure 6C), which may be the main reasons for increased ALT and AST in serum. Therefore, ΔppGpp did not cause serious toxicity in the body and exerted high biosafety when applied for cancer immunotherapy.

## Discussion

Overall antitumor effects of VNP20009 and ΔppGpp in the MC38 colorectal cancer model were comparable, providing experimental basis for the extensive use of ΔppGpp in the treatment of various tumor models. However, ΔppGpp could induce more immune cell infiltration; release high levels of TNF-α, IL-1β, and other antitumor inflammatory factors; and retain high safety profiles *in vivo* compared with VNP20009.

BMCT has been increasingly characterized for its higher tumor specificity, tissue penetration, and lower systemic toxicity over conventional cancer treatments, including radiotherapy, chemotherapy, and surgical resection (Wen et al., 2018). *Salmonella Typhimurium* has been widely used as an excellent strain for BMCT. Previous studies have shown that VNP20009 exhibits excellent antitumor activity in certain types of tumor, and it has entered clinical trials, but the antitumor activity needs to be further improved. The unexpected anticancer efficacy in clinical trials may be due to insufficient bacterial colonization, excessively attenuated bacteria with multiple deletions and SNPs, and obvious adverse effects with increased dose. Therefore, constructing *S. Typhimurium* mutants with high tumor-targeting specificity, deep tissue penetration, and low systemic toxicity is urgently needed for BMCT. ΔppGpp is a mutant strain that drastically increases the half-lethal dose by 1 million-fold in mice (Na et al., 2006; Zheng et al., 2017), allowing the expression of more sufficient anticancer genes or targeted delivery of a higher amount of anticancer drugs and providing better conditions for combinational therapies (Chen et al., 2021a, 2022).

Previous research has shown that *S. Typhimurium* conserves components (such as lipopolysaccharide) on the surface, activates the TLR4 signaling pathway, and causes a series of downstream inflammatory reactions, thereby promoting immune cell infiltration into the TMEs, such as neutrophils, DCs, and macrophages; the immune cells further increase the secretion of some cytokines, such as TNF-α and IL-1β, and reduce immunosuppression in TMEs (Phan et al., 2015; Chen et al., 2017; Zheng et al., 2017). Studies have shown that VNP20009 plays an important role in tumor immunotherapy in combination with other strategies, such as photothermal therapy, chemotherapy, and immune checkpoint blockade (Zhang et al., 2016; Chen et al., 2018). In the present study, ΔppGpp, another *S. Typhimurium* mutant, also exhibited excellent antitumor effects in a mouse colorectal cancer model; it showed less weight loss, higher animal survival, less tissue injury, and higher cytokine production compared with VNP20009. Recently, the combination of *S. Typhimurium* with nanomaterial has yielded exciting results in tumor treatment (Mi et al., 2021), and the integration of molecular imaging techniques has shown high potential of application for tumor diagnosis (Jiang et al., 2013; Xiong et al., 2020). The engineered ΔppGpp shows minimal damage to normal organs *in vivo*, as well as obviously increased animal survival. The drug loading property and *in situ* expression of anticancer payloads make attenuated bacteria as ideal vectors for drug delivery (Chen et al., 2022).

In summary, the engineered ΔppGpp strain manifested less cell invasion and cytotoxicity *in vitro*, higher biosafety, and better therapeutic efficacy on MC38 tumor-bearing mice than VNP20009. ΔppGpp might have greater clinical application potential than VNP20009 in terms of anticancer efficacy and biosafety. Further work should be conducted to optimize the ΔppGpp strain to promote the clinical translation. Subsequently, ΔppGpp-based cargos that carry therapeutic payloads should be developed to stabilize the anticancer immunity.

## Data Availability Statement

The original contributions presented in the study are included in the article/supplementary material; further inquiries can be directed to the corresponding authors.

## Ethics Statement

The animal study was reviewed and approved by the Hunan University Animal Research Committee (Changsha, China).

## Author Contributions

XL and YG conducted the experiments in vitro. XL, YS, YC, and WT conducted the experiments in animal models. XL, YG, and YS were involved in study design and data analysis. XL wrote the Manuscript. JZ and J-JM were responsible for the study design, experiment guidance, and manuscript revision. All authors contributed to the article and approved the submitted version.

## Funding

This work was supported by the Hunan Natural Science Foundation (2020JJ5094), the Changsha Municipal Natural Science Foundation (kq2014061), and the National Research Foundation of Korean Government (MSIT) (2020M3A9G3080282 and 2020R1A5A2031185).

## Conflict of Interest

The authors declare that the research was conducted in the absence of any commercial or financial relationships that could be construed as a potential conflict of interest.

The reviewer [XL] declared a shared affiliation with the authors at the time of the review.

## Publisher’s Note

All claims expressed in this article are solely those of the authors and do not necessarily represent those of their affiliated organizations, or those of the publisher, the editors and the reviewers. Any product that may be evaluated in this article, or claim that may be made by its manufacturer, is not guaranteed or endorsed by the publisher.
